# On Spatiotemporal Overdispersion and Macroparasite Accumulation in Hosts Leading to Aggregation: A Quantitative Framework

**DOI:** 10.3390/diseases11010004

**Published:** 2022-12-27

**Authors:** Jomar Fajardo Rabajante

**Affiliations:** Institute of Mathematical Sciences and Physics, University of the Philippines Los Baños, Laguna 4031, Philippines; jfrabajante@up.edu.ph

**Keywords:** parasitism, aggregation, variance, risk, heterogeneity, social network

## Abstract

In many host–parasite systems, overdispersion in the distribution of macroparasites leads to parasite aggregation in the host population. This overdispersed distribution is often characterized by the negative binomial or by the power law. The aggregation is shown by a clustering of parasites in certain hosts, while other hosts have few or none. Here, I present a theory behind the overdispersion in complex spatiotemporal systems as well as a computational analysis for tracking the behavior of transmissible diseases with this kind of dynamics. I present a framework where heterogeneity and complexity in host–parasite systems are related to aggregation. I discuss the problem of focusing only on the average parasite burden without observing the risk posed by the associated variance; the consequences of under- or overestimation of disease transmission in a heterogenous system and environment; the advantage of including the network of social interaction in epidemiological modeling; and the implication of overdispersion in the management of health systems during outbreaks.

## 1. Introduction

In studying systems at the macroscale, often we use the average or mean dynamics [1,2]. There are advantages in doing this in terms of efficiency and computational tractability, especially if the average dynamics are too pronounced and minute deviations from the average seem negligible. However, there are systems where if we miss important details related to the variance and if we do not observe the dispersion in the dynamics, it could be dangerous or misleading. One case is related to infectious diseases, in which superspreading events are reflected not on the average dynamics but on the overdispersed distribution of disease transmission in the host population [2,3,4]. A superspreading event happens when a disease, such as COVID-19, is transmitted to many secondary cases more than what is expected [5]. In macroparasite infections, hosts with a high parasite burden can be a superspreader of more parasite infections in the host population [6].

In systems theory, we often deal with deterministic approaches. However, such approaches might oversimplify the investigation of the system and often neglect to consider heterogeneity in the environment and in the interaction among actors in the environment. Environmental heterogeneity and the dynamic behavior of parasite transmission can lead to overdispersion, and overdispersion is physically characterized in parasite aggregation or in superspreading events during epidemics [6]. In host-parasite systems, parasite aggregation is usually considered an ecological ‘law’ because it is very common in nature, especially if we consider macroparasites such as worms and ticks [7,8,9]. This aggregation or clustering of parasites in few hosts has an impact on statistical sampling (which means that the normal distribution cannot be used), in estimating disease burden in the population, and in designing mass or targeted interventions. 

There are several studies that highlight the importance of spatiotemporal overdispersion in real-world systems, its causes, and its impact in the emergence of special patterns [10,11,12]. For example, the aggregation of Acanthocephalan parasites in fish populations is hypothesized to be due to spatial random foraging of fish [4]. Even if the parasites are uniformly distributed in a lake, spatial random foraging of fish can still result in parasite aggregation. The resulting pattern shows that only few hosts have a high parasite load, in which they can be superspreaders of parasite eggs in the lake. 

Various factors are involved in parasite load overdispersion and aggregation; the stochasticity and complexity in host–parasite dynamics [13], the heterogeneity in host and parasite populations [6], and the spatiotemporal changes in the environment [4] are some of them. While overdispersion in infectious disease systems has been extensively explored [6,14,15], integrating some of the factors and associated computational techniques may be lacking. Evidently, the number of mathematical and computational papers considering aggregation in their dynamics is very few (e.g., in Susceptible-Infectious-Removed systems) [2,16]. Here, I present a framework behind parasite aggregation in spatiotemporal epidemiological systems. I also present a computational investigation for tracking the dynamics of such systems. This framework, alongside other available techniques [11,17,18,19], could help researchers and practitioners in determining possible causes of clustering in the parasite population as well as in tracking disease burden in the host population. 

## 2. Framework

**Figure 1:**
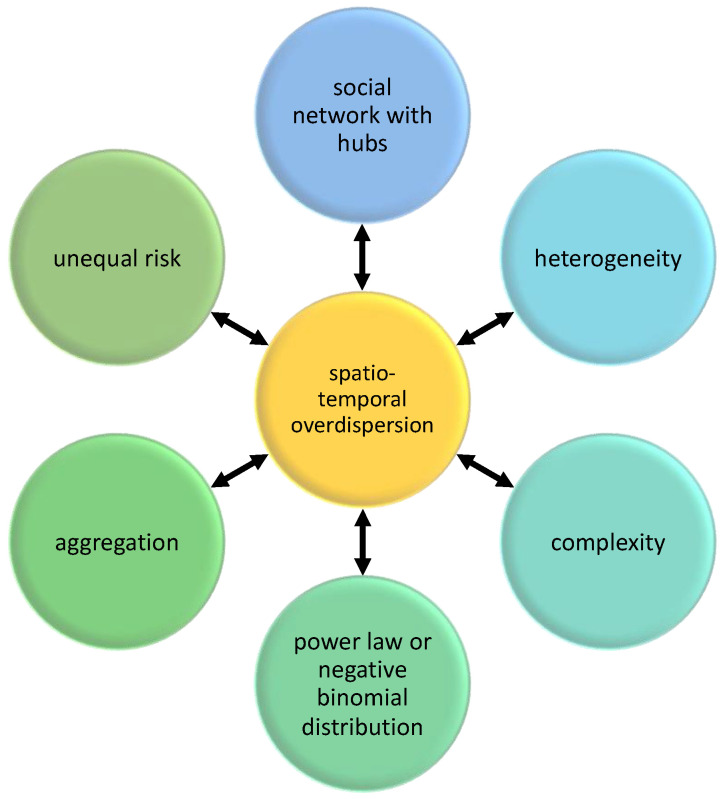
Concepts related to spatiotemporal overdispersion (see Table 1 for the glossary). Each factor can be sufficient to drive parasite load overdispersion in hosts. Social network with hubs, heterogeneity in the characteristics of actors (e.g., hosts), and complexity in host–parasite dynamics may lead to a distribution with variance greater than the mean. This distribution can characterize parasite aggregation in the host population. This aggregated pattern may lead to differential risk, such as superspreading events. These concepts, when investigated, can provide insights about the level of spatiotemporal overdispersion in the system and when tracked, can aid in identifying strategies for infectious disease prevention or control. Overdispersion is the link among concepts presented here. For example, Heterogeneity and Aggregation are both linked to Overdispersion.

In this section, a proposed framework of spatiotemporal overdispersion in epidemiology is presented. The framework consists of relationships among social network, power law distribution, negative binomial distribution, heterogeneity, complexity, aggregation, and risks in host–parasite systems (Figure 1). These concepts are related to the study of systems with spatiotemporal overdispersion leading to parasite aggregation, and hence, I propose that future studies should consider these concepts in their theoretical, experimental, and computational frameworks [3,13,20,21]. Overdispersion is the link among the concepts presented here. In many computational or experimental studies, overdispersion is detected first (variance is greater than the mean) because this is apparent in the data, before mechanisms and outcomes are identified (e.g., aggregation).

Network Science is one of the areas usually used in systems dynamics research. It is now widely known that real networks are not Uniform nor Poisson (randomly) distributed [22,23]. That is, the edge connection between the nodes is not randomly constructed in the mathematical or statistical sense, which implies that the mean of the degree distribution is not equal to its variance. One of the more appropriate distributions is to use a long-tailed distribution, such as the Pareto and power law [17,22]. In many instances, the variance could be large or could be infinite so that the power law distribution can fit. This can be the case in social networks where a special person acting as a node in the network has too many edge connections (referred to as the hub), which can lead the degree distribution to have a huge variance. In epidemiology, social contact matrices used in infectious disease modeling can exhibit this distribution [24,25]. As such, interactions among age groups in various settings (home, work, and school) show heterogeneity that leads to disease transmission with a variance greater than the mean transmission level. In diseases, such as COVID-19, this can be the case as shown by superspreading events [26]. This is an example of spatiotemporal overdispersion, as characterized by the social network (e.g., from contact tracing reports), that can have a significant impact on epidemiological or public health systems, especially during an outbreak [25]. With this, it is implied that nodes with a high degree can be superspreaders and can be targeted to minimize the risk of further spread of infectious diseases.

**Figure 2:**
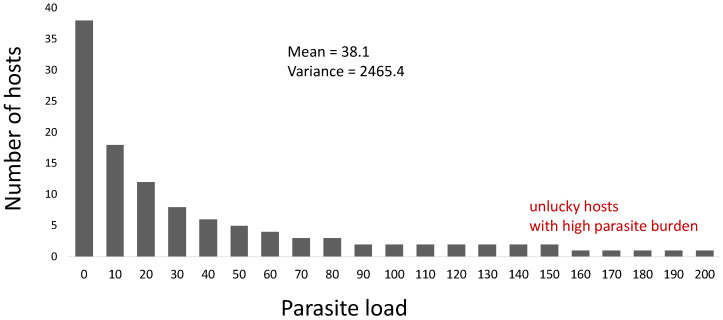
Example distribution where there is parasite aggregation in host population. Variance > Mean. In this distribution, many have zero or low parasite burden, and only a few have high parasite burden.

In the field of parasitology, it is generally considered that parasite aggregation in a host population is an ecological ‘law’ [8]. This is because most host–macroparasite interactions in nature exhibit such a pattern [14]. This parasite aggregation characterizes a distribution where many hosts harbor few or none of the parasites, but a small number of the hosts have a high parasite load (Figure 2). This clustering behavior where only few individual hosts carry a large number of parasites can be due to many reasons. One is due to heterogeneity in the characteristics of the individual hosts, heterogeneity in the parasite population, or heterogeneity in the environment [27]. Indeed, it is expected that if there are heterogeneities, the possibility of having a variance greater than the mean (in terms of the parasite load) is larger [6]. With this kind of variance, it is expected that some hosts harbor more parasites than other hosts. However, how huge should the heterogeneity be to have such ‘variance > mean’ behavior? There are mathematical models and agent-based simulations showing that even if the hosts share similar characteristics, the localities in the environment are homogenous, and the parasites are distributed equally in the environment; even still, food-borne parasite aggregation can arise if the foraging behavior of the host population is random [3,4]. This is possible because of a second reason, which is having a complex parasite life cycle (e.g., with feedback loops) [4,16,28,29].

A minute difference in the spatiotemporal behavior of hosts can be magnified to drive parasite aggregation because of complexity. Parasite aggregation, specifically for macroparasites, arises due to the complex life cycle of the parasites. For example, parasite (e.g., worms) eggs are scattered in an environment (e.g., lake). Intermediate hosts (e.g., zooplanktons) in the environment carry the eggs; these eggs do not grow without the main hosts (e.g., fish). Since the intermediate hosts are a staple food of the main hosts, the main hosts eat them, possibly not knowing the parasite eggs are present. Then, the parasites grow in the main host. After some time, the mature parasites lay eggs, and these eggs are discharged in the excretion of the main hosts. Without proper sanitation, these eggs can contaminate the environment, and then the life cycle of the parasite population repeats. This complex life cycle can be a form of differential feedback loops in a complex system. It is hypothesized that the distribution of parasite aggregation (e.g., as shown in Figure 2) can have a longer tail over time (e.g., more aggregation as the host ages) or as the food chain becomes more convoluted similar to the concept of bioamplification or biomagnification [4,30]. 

The distribution characterized by parasite aggregation may not necessarily be heavy or long-tailed. In most cases, the negative binomial distribution is used, and in some cases, a zero-inflated distribution can be useful in statistical studies [31,32]. There are common metrics that are being calculated to describe and track parasite aggregation. Examples are [15]:

- Variance-to-mean ratio, where if this ratio is approximately equal to 1, then a Poisson (random) distribution could characterize the distribution of parasites in the host population. If it is greater than 1, then parasite aggregation may occur. Smaller values (<1) may represent a distribution following the binomial distribution; if the value is near zero, the parasite distribution could be uniformly or evenly distributed. The variance-to-mean ratio is related to the index of dispersion (D), which can be described by the following [6,23]:
  (1) $$D=\frac{\sigma^{2}}{\mu }\left(n-1\right)$$
  where μ is the mean, and $\sigma^{2}$ is the variance of the distribution of parasites in the host population. The parameter n is the number of sampled hosts.

- Negative binomial parameter k, which can be described as
  (2) $$k=\frac{\mu^{2}}{\sigma^{2}-\mu }.$$
  If $\sigma^{2}\gg \mu$, then k is small or could be near zero, in which parasite aggregation may occur. If $\sigma^{2}\approx \mu$ or k is large, a Poisson (random) distribution may arise [4,6]. This metric is one of the most preferred aggregation metrics.

- Taylor’s Power Law b, in which b is the regression slope described by the following [6,17]:
  (3) $$\sigma^{2}=a+\mu^{b}\;\mathrm{or}\;\log\left(\sigma^{2}\right)=\log\left(a\right)+b\;\log\left(\mu \right)$$
  Here, a and b are fitted against the collected data. The distribution of the parasites in the host population could be uniform if b is zero, random if b is approximately equal to 1, and aggregated if b is significantly greater than 1.

The coefficient of variation is not usually used as an aggregation metric because it is scale-invariant. This means that two distributions—say one with a low total parasite count and the other with a high total parasite count—could reflect similar coefficients of variation. In parasitology, actual counts matter, especially that the parasite load is related to superspreading events and affects the health of the host. Comparing the parasite distribution to the Poisson distribution or to the negative binomial can also be performed using statistical techniques, such as goodness-of-fit tests [33]. 

In compartmental modeling, such as in Susceptible-Infectious-Removed (SIR) framework [34], heterogeneity is represented by incorporating the k parameter in the differential equation model. For example, we can represent the change in the S population due to the disease by [2]:

(4) $$\frac{dS}{dt}=-k\ln\left(1+\frac{\beta I}{kN}\right)S$$

where β is the frequency-dependent transmission rate; I is the population size of the infectious, and N is the total population size. The parameter k is also used in calculating the outbreak threshold (T), which is the number of cases needed for an outbreak to thrive with a probability equal to 1−c. The outbreak is calculated during the initiation phase of the outbreak. It can be estimated using the following formula [35]:

(5) $$T=-\frac{\log\left(c\right)}{\log\left(R_{0}\right)}\left(0.334+\frac{0.689}{k}+\frac{0.408}{R_{0}}-\frac{0.507}{kR_{0}}-\frac{0.356}{R_{0}{}^{2}}+\frac{0.467}{kR_{0}{}^{2}}\right).$$

where $R_{0}$ is the basic reproduction number. 

The reproduction number R (basic or effective) represents the average contagiousness or transmissibility of a disease, while k considers the variance. Both metrics are essential to be reported, not just the famous R. In a disease network, R can be calculated by taking the mean of the number of individuals infected by an infectious host. As we know, the number of secondary cases is often not uniform, and thus, variance is not zero. The variance is important to be reported so that we can gauge how superspreading events affect the contagiousness or transmissibility of a disease in a community. Knowing the possible superspreaders may help in the disease cluster detection and in the minimization of delays in reporting and control.

There are other sources of heterogeneity that can cause overdispersion and aggregation of parasites. Age distribution and aging have a role in accumulating parasites through time (e.g., via foraging), which may lead to aggregation [36]. As shown in Figure 1, there are many pathways that could lead to overdispersion and aggregation. Whatever the reasons are, we need to consider the variance and the possible “black swans” or the outliers, which could not be detected by only investigating the average dynamics of the host–parasite interaction. This framework of thinking could have an impact on the management of health systems, which also has socio-economic implications [37,38]. Overdispersion and aggregation pose unequal exposure, vulnerability, and risk to hosts. In some cases, risk sharing could be managed in such a way that the marginalized and overburdened host can recover and survive. An example of risk sharing is that in a farming community with a schistosomiasis outbreak, human hosts (farmers) with low protection should not be too exposed to the disease compared to farmers with access to protective gear. 

## 3. Method

In this section, the method of tracking the disease dynamics with overdispersion is presented. As shown in Figure 1, there are many pathways to parasite aggregation. Hence, monitoring the mechanisms of various pathways can help in tracking overdispersion leading to aggregation. Social network with hubs, heterogeneity in the characteristics of actors (e.g., hosts), and complexity in the interaction among actors may lead to a distribution with variance greater than the mean. This statistical distribution can characterize aggregation, such as parasite aggregation in host population. This aggregated pattern may lead to differential risk, such as superspreading events. These pathways are affected not just by the biological characteristics of hosts and parasites, but also by environment (including abiotic factors and geographic locality) affecting the host–parasite system [4,30]. These pathways when investigated individually and as part of the whole ecosystem can provide insights about the causes and impacts of the disease dynamics and when tracked can aid in identifying strategies for infectious disease prevention or control.

The reproduction number R and the overdispersion coefficient k are, in practice, not easy to calculate due to a lack of available data (e.g., incomplete contact tracing data). One type of commonly available data is the spatiotemporal disease counts, in which there are methods to approximate R and k [39,40]. 

Following the framework in Figure 1, here are some questions to be asked that can be used in our monitoring:

1. What is the degree distribution of the contact or interaction network (e.g., from contact tracing)?
2. What are characteristics of the hosts (e.g., age, sex, body size, and foraging behavior)?
3. What are the characteristics of the parasites (e.g., age, body size, and complex life cycle)?
4. What are the characteristics of the environment (e.g., microlocality, mixing dynamics, temperature, and food distribution)?
5. What are the characteristics of the host–parasite interaction (e.g., preferential attachment and host immunity)?
6. What is the spatiotemporal parasite load of sampled hosts?

**Figure 3:**
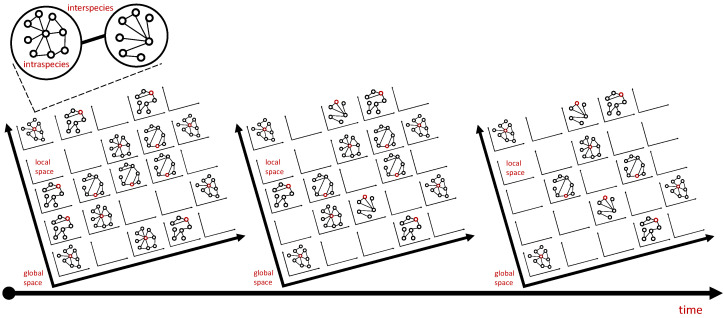
Multiscale investigation of overdispersion. At the top part of the figure, overdispersion can happen at the species population network and also at the interspecies (or intercommunity) network. Food chain or web, predation, parasitism, and other interactions can characterize interspecies network. At the middle part of the figure, overdispersion can be investigated at a certain locality and at the macroscale (global) spatial level. Spatial investigation can be analyzed using a grid system, depending on the availability of data and on the spatial homo/heterogeneity of the interaction networks. For simplicity, a 2D spatial representation is presented here, but a 3D representation can also be performed. Moreover, spatial overdispersion can be dynamic through time. The distribution of parasites in host population can vary and evolve spatially and temporally.

These questions should be disaggregated temporally and spatially (depending on the preferred spatial granularity, i.e., local, regional, and continental scales). The more granular the dataset, the more chance of seeing heterogeneous properties, which means that we need to look at a high-dimensional dataset (as characterized in Figure 3).

**Figure 4:**
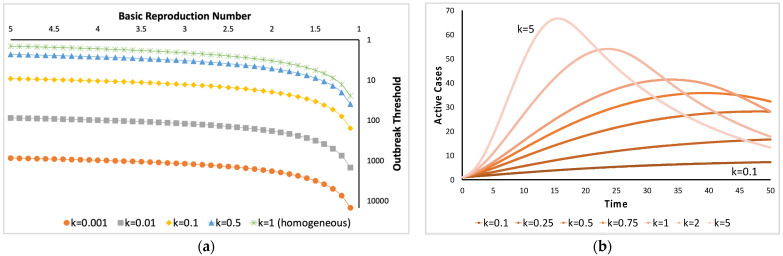
Effect of heterogeneity and overdispersion in epidemics. In both figures, k near zero represents more heterogeneity and high overdispersion. (**a**) 90% probability, k=1 is assumed to be equivalent to a homogeneous transmission; (**b**) k→∞ represents random transmission (e.g., Poisson-distributed spread of infectious disease).

Heterogeneity can affect the monitoring of disease incidence as in the extreme case, it can lead to underestimation of disease transmission due to difficulty in obtaining samples in an overdispersed distribution. However, overestimation can happen when we assume a homogeneous system. Realistic transmission values likely occur between heterogeneous and homogeneous situations. Figure 4 shows the effect of differential k values in epidemiological reports [41].

**Figure 5:**
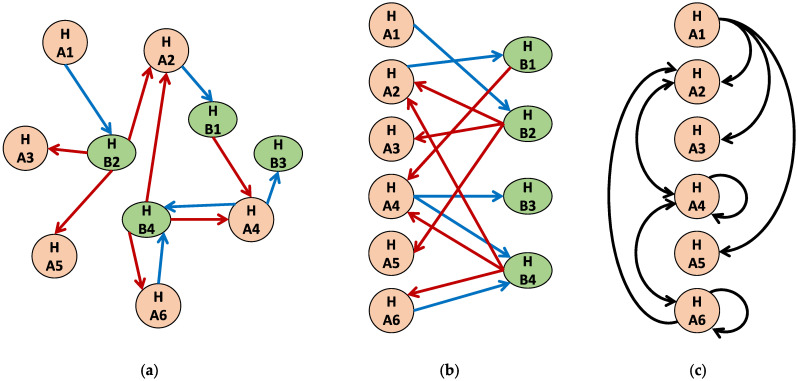
Interaction network among hosts. Blue line denotes transmission from Host species A to Host species B (e.g., parasite eggs from main host to intermediary host). Red line denotes transmission from Host species B to Host species A (e.g., parasite larva from intermediary host to main host). The lines can also be weighted based on volume of parasite loading. (**a**) sample network; (**b**) network is converted to a bipartite graph; (**c**) graph projection where black lines denote transmission interaction via an intermediate host. Note that a multipartite (e.g., tripartite) interaction network can also be studied to account for the role of the parasites.

Question 1 will provide us an idea if there are hubs in the interaction network. If there are intermediate hosts in the interaction, we can look at the network projection (see Figure 5). Questions 2 to 5 will provide us information about the level of heterogeneity in host and parasite populations as well as in the environment. We can obtain many insights from these datasets, such as what characteristics can lead to a more aggregated parasite population. For the monitoring, the spatiotemporal descriptive statistics of the datasets gathered as answers to Questions 1 to 6 can be used to investigate heterogeneity, overdispersion, and aggregation.

Using the familiar equation for risk: risk=hazard×exposure×vulnerability, we can track the unequal risk distribution in a host population due to parasite aggregation. Force of infection (as risk) = disease hazard (which can be characterized by the disease prevalence and degree of environmental favorability for parasites to reproduce) × exposure (which can be characterized by the degree distribution in an interaction network that is affected by the behavior of the host and life cycle of parasite) × transmissibility (which can be characterized by the biophysical properties of the parasites and hosts essential for efficient and effective transmission). The force of infection, thus, is not just a single number or constant, but a distribution of values pertaining to the spatiotemporal dynamics of the host–parasite system. The hazard is dynamic as prevalence changes overtime due to more hosts being infected or due to the removal of hosts (e.g., death or recovery). Exposure is also dynamic based on the location and situation and parasite distribution in the environment. The transmissibility also depends on the host body, which can be infected or not, depending on available immunity or protection [42], and depending on the efficiency of the route of transmission. 

**Figure 6:**
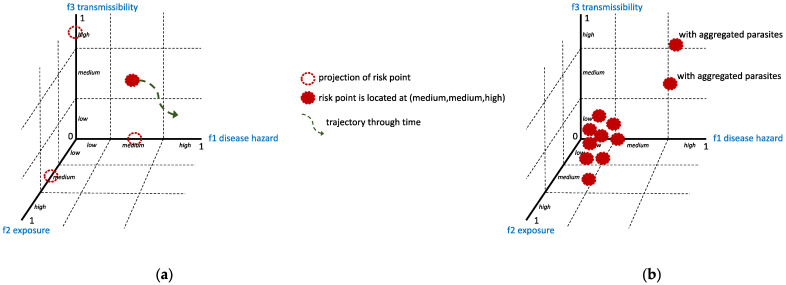
Force of infection as risk is represented as a triple (f1, f2, and f3) meaning value of disease hazard, value of exposure, and value of transmissibility. (**a**) example of a risk point, which characterizes status of one individual host for a specific time frame; (**b**) collection of risk points, which characterizes host population.

After gathering datasets, we grouped the hosts based on the factors affecting the force of infection. In this grouping, heterogeneity and overdispersion are considered. Force of infection is represented as a triple (f1, f2, and f3) meaning value of hazard, value of exposure, and value of transmissibility, see Figure 6. We can consider thresholds as a benchmark, say thresholds for considering low, medium, and high values. For example, the status of one individual host for a specific time frame represents a point in the force of infection space as medium, medium, and high due to medium disease prevalence in the community, medium exposure because the host is sometimes exposed to the environment with parasites, and high transmissibility due to the absence of available protection, respectively (refer to Figure 6 for illustration). Values can be normalized to optimize consistency in setting values so that the risk values are comparable. Moreover, the severity of impact to the health of the host is not yet included in the triple (f1, f2, and f3). A fourth dimension can be included to include severity, which can help in tracking possible hospitalizations.

As an example, consider the simulated datasets in [4]. The dataset is based on the constructed visual agent-based simulation model involving fish hosts in a closed freshwater environment (lentic ecosystem) that forage on zooplanktons harboring macroparasites. In this simulation, several scenarios were considered, such as (i) the initial sizes of the population of both the fish and zooplanktons have a minimal effect on the aggregation of parasites; (ii) increasing the probabilities of reproduction of both fish and zooplankton lead to parasite aggregation among fish hosts; and (iii) aggregation occurs either by decreasing the size of the infection area or increasing the size of the zooplankton-free area in the lentic ecosystem. Relating the simulated datasets to the framework presented here, we can characterize the f1 (in the force of infection triple) based on the density of the parasites in the lentic ecosystem. The value of exposure (f2) can be characterized by the distribution of the parasites in an area (e.g., infected zooplanktons are concentrated only in a limited area; hence, not all fish can be exposed for a certain period of time). The transmissibility (f3) in the simulated datasets is set to be medium to high, i.e., without protection, but there is the possibility of treatment. As shown in the figures in [4], ‘sliders and buttons’ in the NetLogo program can be used to determine the setting of the host–parasite dynamics, which can also determine the (f1, f2, and f3) triple. Different settings could result in different parasite load distributions. 

## 4. Conclusions

In this paper, I discussed a framework based on the mechanisms that can lead to parasite aggregation. Parasite aggregation is a result of overdispersion and heterogeneity, and this aggregated pattern is very common in nature. Thus, it is important not to focus only on the average parasite burden in hosts because parasite aggregation poses a high risk to hosts with a severe parasite load. These hosts can also be potential superspreaders.

In terms of biodiversity, parasite aggregation is beneficial to both macroparasite and host populations (except for few sacrificial hosts) as parasites can have enough population size without exterminating the whole host population. Parasite aggregation could be an example of a collective behavior (e.g., as a result of the complex life cycle of parasites and the dynamic interaction with foraging hosts) with or without the intention of each individual parasite to aggregate. The emergence of this pattern is based on many pathways as shown in Figure 1, which in many circumstances could be part of a host–parasite evolution and a driver to stabilize ecological systems. 

**Figure 7:**
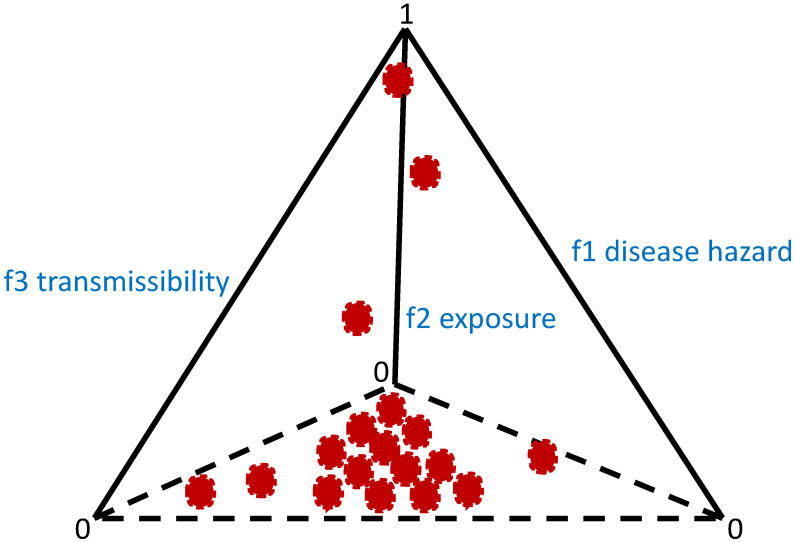
Simplex of risk points that could characterize host population with parasite aggregation. High-risk individual hosts could have high parasite burden. Each red dot could represent an individual host.

One way to investigate heterogeneity is to include the network of interaction in host–parasite systems, which is expected to be non-Poisson. Heterogeneity can lead to differential exposure and vulnerability and to overdispersed risks [26]. We can expect that many host–parasite systems exhibit a pattern similar to Figure 7, where we can observe a 3-simplex of risk points in the force of the infection space. In the simplex, many hosts have minimal risks, but a few have very high risks, which is a sign of parasite aggregation in hosts [27].

Future research directions include determining more pathways or mechanisms that could lead to parasite aggregation. The proposed method of tracking spatiotemporal parasite aggregation can be utilized to investigate datasets from observational studies and to identify trends in parasite aggregation (e.g., to monitor stable equilibrium, seasonalities, and trend anomalies). The proposed integrated framework and method can aid studies related to mammalian parasitic infections, health, zoonoses, and neglected tropical diseases.

## Figures and Tables

**Table 1:** Glossary of terms related to parasite aggregation. These terms, while related to each other, should not be used interchangeably.

Term	Definition
Aggregation	Clustering of parasites in few hosts, while many other hosts have few or none.
Complexity	A characteristic of systems with many dynamic and interacting components. The interaction among the components usually results in an emergent behavior. The interaction among components can be modeled using networks.
Heterogeneity	Presence of variability in the system. Variance is not zero.
Overdispersion	Variance is greater than the mean. Usually, an overdispersed distribution is often characterized by the negative binomial or by the power law.

## Data Availability

Not applicable.
